# Human T-lymphotropic virus 1/2 infection among prisoners of a major penitentiary complex of Goiás State, Central-West Brazil

**DOI:** 10.3389/fpubh.2024.1379237

**Published:** 2024-04-19

**Authors:** Michele Tiemi Okita, Marcos André de Matos, Nara Rúbia de Freitas, Ágabo Macedo da Costa e Silva, Juliana Menara de Souza Marques, Thaís Augusto Marinho, Márcia Alves Dias de Matos, Regina Maria Bringel Martins

**Affiliations:** ^1^Institute of Tropical Medicine and Public Health, Federal University of Goiás, Goiânia, Brazil; ^2^Faculty of Nursing, Federal University of Goiás, Goiânia, Brazil

**Keywords:** HTLV, prisoners, prevalence, high-risk population, Brazil

## Abstract

**Introduction:**

Studies on human T-lymphotropic virus 1/2 (HTLV-1/2) infection are scarce in incarcerated population. Therefore, this study estimated the prevalence of HTLV-1/2 infection among prisoners of the major penitentiary complex of Goiás State, Central-West Brazil, comparing it with available data from other Brazilian regions.

**Methods:**

A cross-sectional study was conducted with 910 prisoners of the major penitentiary complex in the State of Goiás, Central-West Brazil. All participants were interviewed, and their serum samples were tested for anti-HTLV-1/2 using an enzyme-linked immunosorbent assay (ELISA; Murex HTLV-I + II, DiaSorin, Dartford, UK). Seropositive samples were submitted for confirmation by a line immunoassay (INNO-LIA HTLV I/II, Fujirebio, Europe N.V., Belgium).

**Results:**

The majority of participants were males (83.1%), between 25 and 39 years old (56.1%; mean age: 31.98 years), self-reported brown ethnicity (56.2%) and reported 9 years or less of formal education (41.4%). Most reported using non-injectable illicit drugs and various sexual behaviors that present risk for sexually transmitted infections (STIs). The prevalence of anti-HTLV-1/2 was 0.33% (95% CI: 0.07–0.96), HTLV-1 (0.22%) and HTLV-2 (0.11%). The two HTLV-1 seropositive prisoners reported high-risk sexual behaviors, and the HTLV-2 seropositive individual was breastfed during childhood (> 6 months) by her mother and three other women.

**Conclusion:**

These data revealed a relatively low seroprevalence of HTLV-1/2 in prisoners in Central-West Brazil, and evidence of HTLV-1 and HTLV-2 circulation in the major penitentiary complex of Goiás State. Given the prevalence of high-risk sexual behaviors, there is a crucial need to intensify education and health programs in prisons to effectively control and prevent HTLV-1/2 and other STIs.

## Introduction

The human T-lymphotropic virus type 1 (HTLV-1) causes a persistent infection in an estimated 5–10 million people globally, leading to various clinical manifestations. These range from severe conditions like adult T-cell leukemia/lymphoma (ATLL) to HTLV-1-associated myelopathy/tropical spastic paraparesis (HAM/TSP) and other inflammatory diseases (1). Regarding HTLV-2, it is estimated that 800,000 people globally are infected with this virus, which is prevalent in people who inject drugs and American indigenous populations, particularly in Amazon region of Brazil (2, 3). Although HTLV-2 has been associated with rare cases of myelopathy, its pathological role is still unclear (4, 5). Despite these clinical and epidemiological differences, both viruses are transmitted by unprotected sexual intercourse, injecting drug use or transfusion/transplantation of contaminated blood/tissue or organ, and from mother to child, mainly through breastfeeding (1, 5).

There are more than 10.77 million people living in prison worldwide, and the world prison population may well be in excess of 11.5 million (6). Brazil has the third largest prison population in the world (835,643 in December 2022) (7). Incarcerated individuals frequently endure precarious conditions. These include overcrowded cells, inadequate ventilation and lighting, poor infrastructure, and limited access to basic sanitation. Additionally, issues like malnutrition, illicit drug use, and restricted access to health services are prevalent (8, 9), and create an environment conducive to the spread of infectious diseases (10), including bloodborne and sexually transmitted infections (STIs) (11, 12).

This study, recognizing the significant vulnerability of incarcerated individuals and the limited research on HTLV-1/2 in Brazilian prisons (13–17), aims to investigate the prevalence of HTLV-1/2 infection in Goiás State’s major penitentiary complex, Central-West Brazil, and compare these findings with prevalence data and related studies from other regions in Brazil.

## Methods

### Study population

A cross-sectional study was conducted in prisoners of the major penitentiary complex of Goiás State, located in the metropolitan region of Goiânia (capital of the State of Goiás), Central-West Brazil. The study was conducted among prisoners in four units of the Prison Complex of Aparecida de Goiânia in Goiás State, Brazil. These units included the Coronel Odenir Guimarães Penitentiary, Consuelo Nasser Women’s Penitentiary, Provisional Prison House, and Triage Center.

Sample size was calculated based on an estimated HTLV-1/2 infection prevalence of 1.3% among Brazilian prisoners (13, 14, 16), a 95% confidence level (α < 0.05), 80% statistical power (*β* = 20%), a 1% precision, and a design effect of 1.5%. This calculation yielded a minimum sample size of 740 participants, therefore the study included 910 prisoners.

From May 2018 to January 2020, all prisoners were invited to participate in this study, and they were recruited with the help of prison security agents and with the assistance of inmate health monitors, who were responsible for managing health-related requests in the prisons. Prisoners aged 18 years or older were included in the study. Individuals who showed aggressive behavior and those who appeared to be under the influence of psychotropic drugs were excluded from the study.

### Ethical aspects and sample collection

This study was approved by the Research Ethics Committee of the Clinical Hospital, Federal University of Goiás (reference number 80757617.9.1001.5078). All participating subjects provided their voluntary agreement by signing the free and informed consent form.

Data and blood samples were collected at a private location in each unit of the Prison Complex of Aparecida de Goiânia. All participants were previously informed about the aims of the study, and those who agreed to participate signed the informed consent form. A structured and comprehensive questionnaire was employed to gather data on participants’ sociodemographic characteristics, risk behaviors for HTLV-1/2 infection, and medical history. Then, a sample of venous blood (10 mL) was collected from each participant for laboratory tests.

### Laboratory tests

Serum samples from all participants were tested for anti-HTLV-1/2 (Murex HTLV-I + II, DiaSorin, Dartford, UK) by enzyme-linked immunosorbent assay (ELISA). Seropositive samples were confirmed using a line immunoassay (INNO-LIA HTLV I/II, Fujirebio, Europe N.V., Belgium). Briefly, this assay uses strips containing four non-typed-specific antigens (two gag: p19 I/II, p24 I/II; and two env: gp46 I/II and gp21 I/II) for anti-HTLV confirmation. Additionally, three typed-specific antigens for HTLV-1 (gag p19-I and env gp46-I) and for HTLV-2 (env gp46-II) are used for differentiation of HTLV-1 and HTLV-2 antibodies. Samples that tested anti-HTLV positive by LIA (reactive with at least two of the following confirmation bands: p19 I/II or p24 I/II or gp46 I/II and gp21 I/II) were subsequently considered positive for anti-HTLV-1 (if the intensities of p19-I and gp46-I bands were higher than that of the gp46-II band) or anti-HTLV-2 (if the gp46-II band was more intense than the p19-I and gp46-I bands). All assays were conducted according to the respective manufacturer’s instructions.

Anti-HTLV-1/2 positive samples were tested for human immunodeficiency virus 1 (HIV-1) co-infection using a four-generation ELISA for the simultaneous detection of HIV-1 p24 antigen and anti-HIV-1/2 antibodies (HIV Ag/Ab ELISA 4a Generation test, Wiener Lab, Rosario, Argentina); hepatitis B virus (HBV) co-infection using an ELISA for hepatitis B surface antigen (HBsAg, Biokit S.A., Bioelisa, Spain); and for hepatitis C virus (HCV) exposure using an ELISA for antibodies to the HCV (anti-HCV, Bioelisa-Bioclin®, Quibasa, Brazil).

### Data analysis

Data were analyzed using the IBM Statistical Package for the Social Sciences (SPSS) (IBM SPSS Statistics for Windows, Version 20.0). Descriptive analyses were performed using frequency distributions, mean values, and standard deviations. Prevalence of anti-HTLV-1/2 was estimated using a confidence interval of 95% (95% CI).

## Results

Table 1 details the sociodemographic characteristics of the study participants. The majority were male (83.1%), self-identified as heterosexual (93.4%), aged between 25 and 39 years (56.1%; mean age of 31.98 ± 9.69 years), and of brown ethnicity (56.2%). Nearly half were single (49.3%), with 41.4% reporting 9 years or less of formal education.

**Table 1:** Demographic characteristics of the prison study population.

Variable	*N* = 910	%
Age (years)		
18–24	218	24.0
25–39	511	56.1
40–59	169	18.6
≥ 60	12	1.3
Gender		
Male	756	83.1
Female	154	16.9
Sexual orientation (self-declared)		
Heterosexual	850	93.4
Bisexual	32	3.5
Homosexual	22	2.4
Other	6	0.7
Ethnicity (self-reported)		
Brown	512	56.2
White	217	23.9
Black	149	16.4
Asian	24	2.6
Indigenous	8	0.9
Marital status		
Single	449	49.3
Married/living with partner	374	41.1
Divorced/widowed	87	9.6
Education		
Illiterate	11	1.2
≤ 9 years	366	40.2
10–12 years	295	32.4
≥ 13 years	234	25.7
Not reported	4	0.5

As shown in Table 2, a minority of prisoners reported receiving blood transfusions (11.8%). While a significant portion used illicit drugs (85.2%), only a small fraction (4.2%) engaged in injecting drug use. High-risk sexual behaviors were common: 75.3% reported more than 10 sexual partners in lifetime; the prevalence of vaginal, oral, and anal intercourse was 98.1, 90.4, and 78.3%, respectively; 83.9% engaged in homosexual intercourse; 77.1% had sex with drug-using partners; and 67.1% with sex workers. Condom use was infrequent or occasional for 85.4% of the participants. Additionally, 37.1% reported group sex, and 32.3% reported sex for money. A history of STIs was noted in 25.7% of participants.

**Table 2:** Behavioral characteristics reported by 910 prisoners of the major penitentiary complex of Goiás State, Central-West Brazil.

Characteristics	*n*/N*	%
History of blood transfusion	107/905	11.8
Ever used any illicit drug	775/910	85.2
Ever used any illicit injecting drug	38/910	4.2
Number of lifetime sexual partners		
1	4/891	0.4
2–5	89/891	10.0
6–10	127/891	14.3
>10	671/891	75.3
Type of sexual intercourse (lifetime)		
Vaginal	892/909	98.1
Oral	822/909	90.4
Anal	712/909	78.3
Ever had homosexual intercourse	755/900	83.9
Ever had sex with a drug user partner	699/907	77.1
Ever had sexual intercourse with a sex worker	609/908	67.1
Ever had group sex	337/909	37.1
Ever had sex for money	294/910	32.3
Use of condom (lifetime)		
Never/Sometimes	772/904	85.4
Always	132/904	14.6
History of STIs	234/909	25.7

^*^n represents the number of individuals who answered yes, and *N* represents the number of individuals who answered the question; STIs, sexually transmitted infections.

Among the 910 prisoners enrolled, 3 (0.33%; 95% CI: 0.07–0.96) were repeatedly anti-HTLV-1/2 positive by ELISA. After confirmatory testing (LIA), two were positive for HTLV-1 (0.22%) and one was positive for HTLV-2 (0.11%). The distribution of anti-HTLV-1/2 by gender revealed a higher seroprevalence among female prisoners (1.3%; 2/154) than among male prisoners (0.13%; 1/756). None of the seropositive individuals reported symptoms associated with HTLV-1/2 infection at the time of interview.

Regarding the seropositive individuals, detailed in Table 3, the two HTLV-1 positive cases included a 55-year-old male (PLG-440) and a 21-year-old female (PLG-611), both reporting unprotected sexual intercourse with multiple partners and a history of STIs. The HTLV-2 positive case, a 69-year-old female (PLG-240), had unprotected intercourse only with her sole partner and a notable history of being breastfed for over 6 months by her mother and three other women. None of the seropositive individuals reported blood transfusions or injecting drug use. In addition, one HTLV-1 positive case (PLG-611) was co-infected with HIV-1, and there were no HTLV-HBV or HTLV-HCV coinfected participants.

**Table 3:** Characteristics of HTLV-1/2-positive prisoners of the major penitentiary complex of Goiás State, Central-West Brazil.

Characteristics	PLG 440	PLG-611	PLG 240
Age (years)	55	21	69
Gender	Male	Female	Female
Ethnicity	White	Brown	Brown
Birthplace	RS	MA	PI
Marital status	Widowed	Single	Married
Education (years)	26	9	5
Previous incarceration	No	No	Yes
Time in prison (months)	96	3	36
Breastfed as a child (> 6 months)	Yes	NR	Yes
History of blood transfusion	No	No	No
Ever used any illicit injecting drug	No	No	No
Sexually active	Yes	Yes	Yes
Type of sexual intercourse	Vaginal, oral, anal	Vaginal, oral	Vaginal
Use of condoms (lifetime)	Sometimes	Sometimes	No
Number of sexual partners (lifetime)	10	10	1
History of STIs	Yes	Yes	No
Type of HTLV	HTLV-1	HTLV-1	HTLV-2
HIV-1 co-infection	No	Yes	No

HIV-1, human immunodeficiency virus 1; MA, Maranhão; PI, Piaui; RS, Rio Grande do Sul; STIs, sexually transmitted infections; NR, not reported.

## Discussion

This study, the second to focus on HTLV-1/2 infection in Central-West Brazil’s prison population, addresses a critical issue in a strategic region. This region’s borders with other Brazilian states, Bolivia, and Paraguay make it a pivotal area for drug trafficking and criminal activities (18). In addition, our data were compared with the limited data available from other Brazilian regions in order to guide prevention actions and successful control of HTLV-1/2 and other infections in prison settings.

The sociodemographic characteristics of the studied population were similar to those reported by Melo Bandeira et al. (17) in prisoners in Mato Grosso do Sul State, Central-West Brazil, such as mostly adults aged 25–39 years, males, self-reported as brown ethnicity, single, and with less than 9 years of formal education.

Few participants reported a history of blood transfusions, with most transfusions occurring after November 1993, the onset of mandatory anti-HTLV-1/2 screening in Brazilian blood banks (19). Despite widespread illicit drug use, injecting drug use was infrequent, both in this study and among other incarcerated individuals (15, 17, 20). The majority engaged in high-risk sexual behaviors, including having multiple sexual partners and engaging in various types of sexual intercourse (vaginal, oral, and anal). Relationships with drug-using partners or sex workers were also common, alongside low condom usage. A quarter of the participants also reported a history of STIs, aligning with findings from other Brazilian prison studies (15–17, 20, 21), reinforcing the alarming vulnerability of incarcerated people to STIs and the importance of screening, treatment, and prevention strategies targeting this specific population.

The observed seroprevalence of HTLV-1/2 in this study (0.33%; 95% CI: 0.07–0.96) was significantly higher than the rate among local blood donors (0.09%) (22). Nevertheless, relative to other Brazilian prison populations (Table 4), this prevalence was similar to that estimated in prisoners in Mato Grosso do Sul State (0.40%; CI 95%: 0.15–0.87) (17) and falls within the confidence interval reported for incarcerated adolescents in Salvador, Bahia (1.01%; CI 95%: 0.21–2.92) (16). In prior studies, however, Broutet et al. (13) observed a higher seroprevalence (1.41%; CI 95%: 0.52–3.03) in Fortaleza, Ceará. Also, Catalan-Soares et al. (14) determined a seroprevalence of 1.59% (95% CI: 0.04–8.53) among male prisoners in Manhuaçu, Minas Gerais, in a study that included a smaller number of participants.

**Table 4:** Prevalence rates of HTLV-1/2, HTLV-1 and HTLV-2 infections among prisoners in Brazil.

Region (city/ state)	Sample size (*N*)	Sample collection year(s)	Prevalence % (CI 95%)			Reference
			HTLV-1/2	HTLV-1	HTLV-2	
Northeast						
Fortaleza/Ceará	427	1993–1994	1.41 (0.52–3.03)	0.47 (0.06–1.68)	0.47 (0.06–1.68)	Broutet et al. (13)
Salvador/Bahia	297	2004–2005	1.01 (0.21–2.92)	–	–	Fialho et al. (16)
Southeast						
Manhuaçu/Minas Gerais	63	1994	1.59 (0.04–8.53)	–	–	Catalan-Soares et al. (14)
Vitória/Espirito Santo	121	1997	–	4.13 (1.35–9.38)	–	Miranda et al. (15)
Central-West						
Mato Grosso do Sul	1,507	2015–2018	0.40 (0.15–0.87)	0.20 (0.04–0.59)	0.13 (0.02–0.48)	Melo Bandeira et al. (17)
Goiânia/Goiás	910	2017–2020	0.33 (0.07–0.96)	0.22 (0.03–0.79)	0.11 (0.00–0.61)	Current study

CI, confidence interval.

Internationally, the HTLV-1/2 seroprevalence in this study was lower than those reported among specific groups of prisoners, such as male inmates in Maryland, USA (0.93%; CI 95%: 0.55–1.47) (23), HIV-infected inmates in Mozambique (1.55%; CI 95%: 0.67–3.04) (24), immigrants in prison in Italy (2.52%; CI 95%: 1.09–4.91) (25), and prisoners in Tijuana, Mexico (7.07%; CI 95%: 4.79–10.00) (26), but a study in a Danish prison reported no cases of HTLV-1/2 infection (27), indicating varied prevalence rates among prisoners globally.

The distribution of HTLV-1/2 infection by gender showed a higher seroprevalence among female prisoners, which was consistent with that reported in Mozambique (24), possibly reflecting the more efficient transmission of virus from male to female during sexual intercourse (28). On the other hand, this finding differs from previous reports in Brazil which found a higher prevalence of HTLV-1/2 in male prisoners, possibly reflecting their high-risk behaviors (16, 17).

In the major penitentiary complex of Goiás State, we detected anti-HTLV-1 (0.22%) and anti-HTLV-2 (0.11%). This finding aligns with previous research in Fortaleza, Ceará, where anti-HTLV-1 (0.47%) and HTLV-2 (0.47%) were found among prisoners (13). More recently, Melo Bandeira et al. (17) also found the presence of HTLV-1 (0.20%) and HTLV-2 (0.13%) in people living in prisons in Mato Grosso do Sul. Relative to other studies on HTLV-1/2 infection in Goiás, HTLV-1 and 2 dual infections were observed only in patients with pulmonary tuberculosis (29), a population among whom one quarter reported previous incarceration.

Both HTLV-1 seropositive prisoners (PLG-440 and PLG-611) reported unprotected sex with multiple partners and history of STIs. These risky sexual behaviors may contribute to the observed seropositivity for this infection among the studied population since unprotected sexual intercourse and a higher number of partners increase the risk of HTLV-1 transmission (1, 28, 30). In addition, one of these individuals (PLG-611) was co-infected with HIV-1. As HTLV-1 and HIV-1 share common transmission routes, co-infection can occur in endemic areas, as reported in other prison populations (17, 24, 25).

Additionally, two seropositive individuals, one with anti-HTLV-1 (PLG-440) and the other with HTLV-2 (PLG-240), reported being breastfed during childhood. Notably, PLG-240 was breastfed for over 6 months by her mother and three other women in Piaui State, a detail that aligns with findings from a previous study on blood donors and their families in Piaui, where long-term breastfeeding during childhood was linked to HTLV-1/2 infection (31). In fact, HTLV-1/2 is transmitted primarily through infected bodily fluids including breast milk (1, 5). As reported elsewhere (32–34), it is important to note cross-breastfeeding as a potential risk to transmit HTLV-1/2 infection in addition to mother-to-child transmission. Therefore, implementing antenatal screening for HTLV-1/2 and interventions to limit or avoid breastfeeding by women living with HTLV-1/2 are essential to prevent the spread of this infection in endemic regions.

This study has some limitations. First, all interviews were performed face-to-face; therefore, some findings are subject to response biases, especially those regarding sexual behaviors/practices, illicit drug use, etc. Otherwise, some strategies were used to minimize potential biases, including previously trained interviewers and a private place for interviews in each unit of the Prison Complex of Aparecida de Goiânia. Also, the exclusion of individuals who appeared to be under the influence of psychotropic drugs from the study may bias the results potentially underestimating the prevalence; however, only a few prisoners were excluded considering this criterion. Due to health restrictions imposed by the COVID-19 pandemic, it was not possible to collect another blood sample from the three anti-HTLV-1/2 positive individuals for HTLV proviral DNA detection, and ultimately the seropositive individuals were transferred to other cities. Despite these limitations, this study provides valuable epidemiological information on HTLV-1/2 in an incarcerated population in Central-West Brazil.

## Conclusion

This study revealed a relatively low seroprevalence of HTLV-1/2 infection in the major penitentiary complex of Goiás State, Central-West Brazil. Importantly, it also indicated the presence of HTLV-1 and HTLV-2 in this setting. The prevalence of high-risk behaviors, particularly sexual behaviors, and the interactions of incarcerated individuals with the external community, underscore the critical need for targeted programs in prisons. These programs should focus on the diagnosis, control, and prevention of HTLV-1/2 and other STIs.

## Data availability statement

The raw data supporting the conclusions of this article will be made available by the authors, without undue reservation.

## Ethics statement

The studies involving humans were approved by Research Ethics Committee of the Clinical Hospital, Federal University of Goiás. The studies were conducted in accordance with the local legislation and institutional requirements. Written informed consent for participation in this study was provided by the participants' legal guardians/next of kin.

## Author contributions

MO: Conceptualization, Writing – review & editing, Methodology. MaM: Conceptualization, Methodology, Writing – review & editing. NF: Methodology, Writing – review & editing, Formal analysis. ÁS: Methodology, Writing – review & editing. JM: Methodology, Writing – review & editing. TM: Methodology, Writing – review & editing. MáM: Writing – review & editing, Conceptualization, Writing – original draft. RM: Conceptualization, Writing – original draft, Writing – review & editing, Supervision.
